# Risk factors for proximal radial abnormalities in children with untreated chronic Monteggia fractures: a review of 142 cases

**DOI:** 10.1186/s10195-024-00793-z

**Published:** 2024-11-29

**Authors:** WenTao Wang, QianQian Mei, Hang Liu, YueMing Guo, HaiBo Mei, Federico Canavese, Antonio Andreacchio, HanQing Lyu, ShunYou Chen, ShengHua He

**Affiliations:** 1 Department of Orthopedics, Shenzhen Traditional Chinese Medicine Hospital, 1st Fuhua Road of Futian District, Shenzhen, 518033 Guangdong China; 2 Fourth Clinical Medical College of Guangzhou University of Chinese Medicine, Shenzhen, Guangdong China; 3 Department of Pediatric Orthopedics, Shenzhen Children’s Hospital, Shenzhen, Guangdong China; 4 Department of Pediatric Orthopedics, Children’s Hospital of Chongqing Medical University, Chongqing, China; 5 Department of Pediatric Orthopedics, Foshan Hospital of Traditional Chinese Medicine, Foshan, Guangdong China; 6 Department of Pediatric Orthopedics, Hunan Children’s Hospital, Changsha, Hunan China; 7 Orthopedic and Traumatology Department, IRCCS Istituto Giannina Gaslini, Via Gerolamo Gaslini 5, Genoa, Italy; 8 DISC-Dipartimento di scienze chirurgiche e diagnostiche integrate, University of Genova, Viale Benedetto XV No 6, Genova, Italy; 9 Department of Pediatric Orthopaedics, Vittore Buzzi Children’s Hospital, 20154 Milan, Italy; 10 Department of Radiology, Shenzhen Traditional Chinese Medicine Hospital, 1st Fuhua Road of Futian District, Shenzhen, 518033 Guangdong China; 11 Department of Pediatric Orthopedics, Fuzhou Second Hospital, 47th Shangteng Road of Cangshan District, Fuzhou, 350007 Fujian China

**Keywords:** Chronic Monteggia fracture, Children, Anatomy, Proximal radius abnormality, Radial head, Risk factor

## Abstract

**Background:**

The risk factors for proximal radial abnormalities (PRA) in paediatric patients with untreated chronic Monteggia fractures (CMFs) are unclear. This multicentre study aimed to evaluate the risk factors for PRA in children with untreated CMFs.

**Materials and methods:**

The clinical data of 142 patients (mean age at the time of injury: 5.73 years) with untreated unilateral CMFs were retrospectively reviewed. The radial neck-shaft angle (RNS_AP_) and radial head size (RH_L_) were measured on anteroposterior (AP) and lateral (L) radiographs, respectively. The RH_L_ size was the ratio of the widest width of the proximal radial metaphysis to the narrowest radial neck width. The En-RNS_AP_ and En-RH_L_ were the ratios of the enlargement (En) of the RNS_AP_ angle and RH_L_ size of the injured elbow to those of the uninjured elbow, respectively. Paired-sample *t*-tests, single-factor analyses and multiple linear regression analyses were performed to evaluate the correlation between the differences in these parameters between the injured and uninjured elbows and the assessed risk factors. These risk factors included institution, sex, laterality, age at injury, time from injury to diagnosis, direction of RH dislocation, distance of RH dislocation (DD-RH), presence of radial or median nerve injury, heterotopic ossification and immobilization of the elbow after injury.

**Results:**

In children with untreated CMFs (mean time from injury to diagnosis: 14.6 months), Student’s *t*-test revealed a significant difference in the RH_L_ size (*P* < 0.001) but not in the RNS_AP_ angle (*P* = 0.075) between the injured and uninjured elbows. Pearson correlation analysis revealed a potential correlation between En-RH_L_ and age at the time of injury (*P* = 0.069), time from injury to diagnosis (*P* < 0.001) and DD-RH (*P* < 0.001), excluding other risk factors (*P* > 0.05). Multiple linear regression analysis revealed that age at the time of injury (*P* = 0.047), time from injury to diagnosis (*P* = 0.007) and DD-RH (*P* = 0.001) were risk factors for an increased En-RH_L_ in patients with untreated CMFs; the variability in En-RH_L_ among the other three risk factors was 21.4%.

**Conclusions:**

In paediatric patients with unilateral untreated CMFs, PRA of the injured elbow consisted mainly of RH enlargement or radial neck narrowing rather than valgus deformities of the proximal radius. Older age at injury, increased time from injury to diagnosis and DD-RH were risk factors for more severe PRA.

**Level of evidence:**

III.

## Introduction

The Monteggia fracture was described as an ulnar fracture at the proximal edge associated with radial head (RH) dislocation [1]. According to the direction of the RH dislocation and the angulation of the ulnar shaft fracture, Jose Luis Bado classified the fracture patterns into four types, including type I (anterior), type II (posterior), type III (lateral) and type IV (RH dislocation in any direction and ulnar and radial shaft fractures) [1].

Approximately 30–50% of Monteggia fractures may be missed in children [2–6]. Acute injuries that are not diagnosed within 4 weeks of trauma can progress to chronic Monteggia fractures (CMFs) [2–6]. Owing to an incongruent radiocapitellar joint and an immature proximal radial physis [2, 4], the pattern of proximal radial development and subsequent proximal radial anomalies (PRA) of the injured elbow in paediatric patients with CMFs has not been elucidated.

Baydar et al. and Stragier et al. reported observing an enlarged RH during surgery for paediatric CMFs [7, 8] however, the inability to compare the size of the RH of the injured side with that of the uninjured side during surgery has made intraoperative observations somewhat inconclusive [7, 8]. Although Kim et al. measured both the size of the RH and the angle of the radial neck-shaft (RNS) of the injured and uninjured sides radiographically in 12 paediatric patients with unilateral CMFs, the lack of statistical analysis in their study made their conclusions unconvincing [9]. These limitations may be explained by the rarity of paediatric CMFs, which account for less than 1% of all paediatric fractures [2, 3]. To our knowledge, due to the lack of sufficient sample sizes and statistical power, there have been no detailed comparisons of the radiographic characteristics of PRA between the injured and uninjured sides of paediatric patients with untreated CMFs.

Neither the development of PRA nor the risk factors for PRA associated with difficult reduction of the RH in pediatric patients with untreated CMFs have been determined [7, 9]. According to several reports, several risk factors, including age at the time of injury and time from injury to diagnosis, are responsible for the failure of anatomical reduction in paediatric patients with surgically treated CMFs, but the reasons why these risk factors led to the failure of surgical treatment were not provided, nor were the potential correlations between these risk factors and the PRA of the injured elbow [10–13]. Therefore, it remains unclear whether PRA of the injured elbow could be considered a direct cause of surgical treatment failure in paediatric patients with CMFs.

Therefore, the aim of this large multicentre study was to compare radiographic measurements of the injured and uninjured sides and then investigate the risk factors for PRA of the injured elbow in paediatric patients with unilateral CMFs. Our hypothesis was that PRA of the injured elbow could be considered a direct cause of surgical reduction failure in paediatric patients with CMFs due to the correlation between PRA of the injured elbow and several risk factors, including age at the time of injury and the time from injury to diagnosis.

## Materials and methods

The study was approved by the institutional review board (no. 2023132), and we retrospectively reviewed the clinical data of 312 paediatric patients with CMFs who were diagnosed more than 2 weeks after trauma. Patients were consecutively treated at four institutions between March 2015 and March 2023.

The inclusion criteria were as follows: (1) diagnosed with a unilateral CMF 4 weeks or more after trauma [4–6]; (2) age 17 years or younger at the time of diagnosis; (3) no history of previous surgical treatment or attempted reduction of the dislocated RH; and (4) complete clinical and radiographic records, including standard preoperative anteroposterior (AP) and lateral full-length forearm radiographs.

Patients who met one or more of the following criteria were excluded: (1) congenital dislocation of the RH, pathologic fracture, concomitant metabolic, neurologic or genetic disorders, or infantile osteoporosis; (2) concomitant fracture of the proximal radial growth plate; or (3) concomitant bilateral traumatic RH dislocation.

A total of 142 of 312 children (45.5%) with unilateral untreated CMFs were included in this study. Patients came from four institutions: 82 (57.7%) from Institution 1, 27 (19%) from Institution 2, 21 (14.8%) from Institution 3 and 12 (8.5%) from Institution 4.

The cohort consisted of 92 (64.8%) males and 50 (35.2%) females. A total of 81 unilateral CMFs (57%) were located in the right elbow, and the remaining 61 (43%) were located in the left elbow.

A total of 74 patients (52.1%) underwent cast or brace immobilization for 3–4 weeks to treat ulnar fractures associated with RH dislocation, and the remaining 68 patients (47.9%) did not. A total of 24 CMFs (16.9%) had an associated diagnosis of radial (*n* = 11; 78.6%) or median (*n* = 3; 21.4%) nerve injury, while the remaining 118 CMFs (83.1%) did not.

A total of 170 patients (54.5%) were excluded for the following reasons: incomplete clinical and radiographic data (*n* = 129; 41.3%), diagnosed less than 4 weeks after injury (*n* = 28; 9%), non-standard radiographs (*n* = 11; 3.5%), diagnosed with congenital dislocation of the RH (*n* = 1; 0.3%) and pathologic fracture (*n* = 1; 0.3%).

According to Wang et al. [14], the direction of RH dislocation can be classified into 5 types based on AP and lateral radiographs: anterior-lateral (*n* = 58; 40.8%), anterior (*n* = 52; 36.6%), lateral (*n* = 7; 4.9%), anterior-medial (*n* = 22; 15.5%) and posterior-lateral (*n* = 3; 2.1%).

According to AP and lateral radiographs of untreated CMFs, heterotopic ossification was confirmed when there was newly formed ectopic bone that was not present at the time of the initial trauma and was not related to the fracture fragments [15, 16]. In this study, heterotopic ossification was present in 15 of 142 elbows (10.6%) and was absent in the remaining 127 (89.4%).

### Radiographic measurements: RNS_AP_ angle and RH_L_ size

According to Kim et al. [9, 17], the severity of PRA is reflected by the difference in the RNS_AP_ angle and the RH_L_ size, which were measured on AP and lateral (L) radiographs, respectively, between the injured and uninjured elbow joints.

**Fig. 1:**
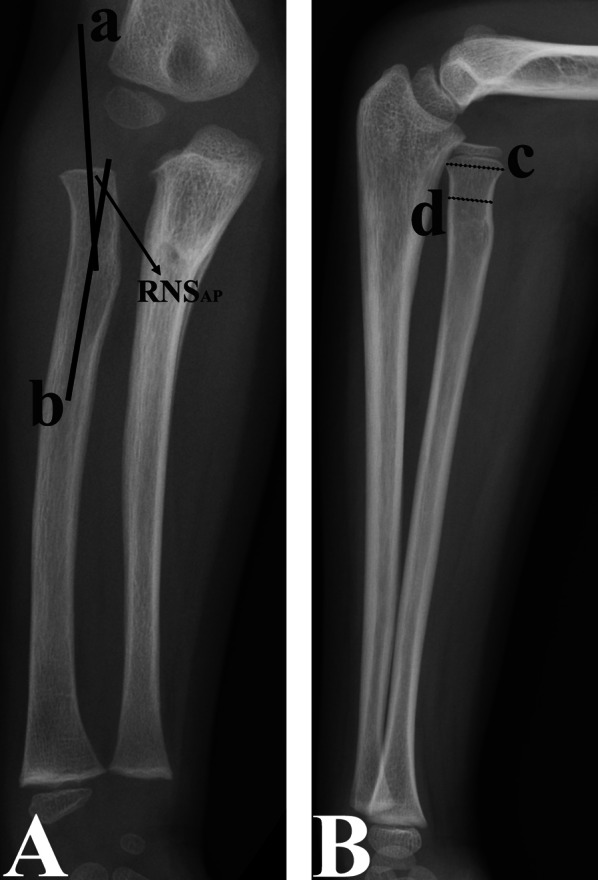
Measurement of the RNS_AP_ angle and RH_L_ size on anteroposterior (**A**) and lateral (**B**) radiographs. RNS_AP_ angle (marked with black arrow): the angle between line a (the radial neck axis) and line b (the proximal radial shaft axis) on the anteroposterior radiograph (**A**); RH_L_ size: the ratio of the width of the widest part of the proximal radial metaphysis (line c) to the narrowest radial neck width (line d) on the lateral radiograph (**B**)

The RNS_AP_ angle was defined as the angle between the axis of the radial neck and that of the proximal radius on AP radiographs [9, 17]; the greater the RNS_AP_ angle, the more severe the valgus deformity of the proximal radius (Fig. 1).

The RH_L_ size was expressed as the ratio of the widest width of the proximal radial metaphysis to the narrowest width of the radial neck on lateral radiographs [9, 17]; as a result, the larger the RH_L_ size, the narrower the radial neck (Fig. 1).

### Radiographic measurements: En-RNS_AP_ and En-RH_L_

En-RNS_AP_ and En-RH_L_ are expressed as the ratio of the enlargement (En) of the RNS_AP_ angle and RH_L_ size of the injured elbow to that of the uninjured elbow, which reflects the differences in the RNS_AP_ angle and RH_L_ size between the injured and uninjured elbows. The increased En-RNS_AP_ and En-RH_L_ showed that the RNS_AP_ angle and RH_L_ size of the injured elbow were larger than those of the uninjured elbow, respectively.

According to the definition of the RNS_AP_ angle and RH_L_ size, a larger En-RNS_AP_ reflects more severe valgus deformities of the proximal radius of the injured elbow, and an increased En-RH_L_ indicates a larger RH_L_ size or narrower radial neck of the injured elbow.

### Radiographic measurements: DD-RH

**Fig. 2:**
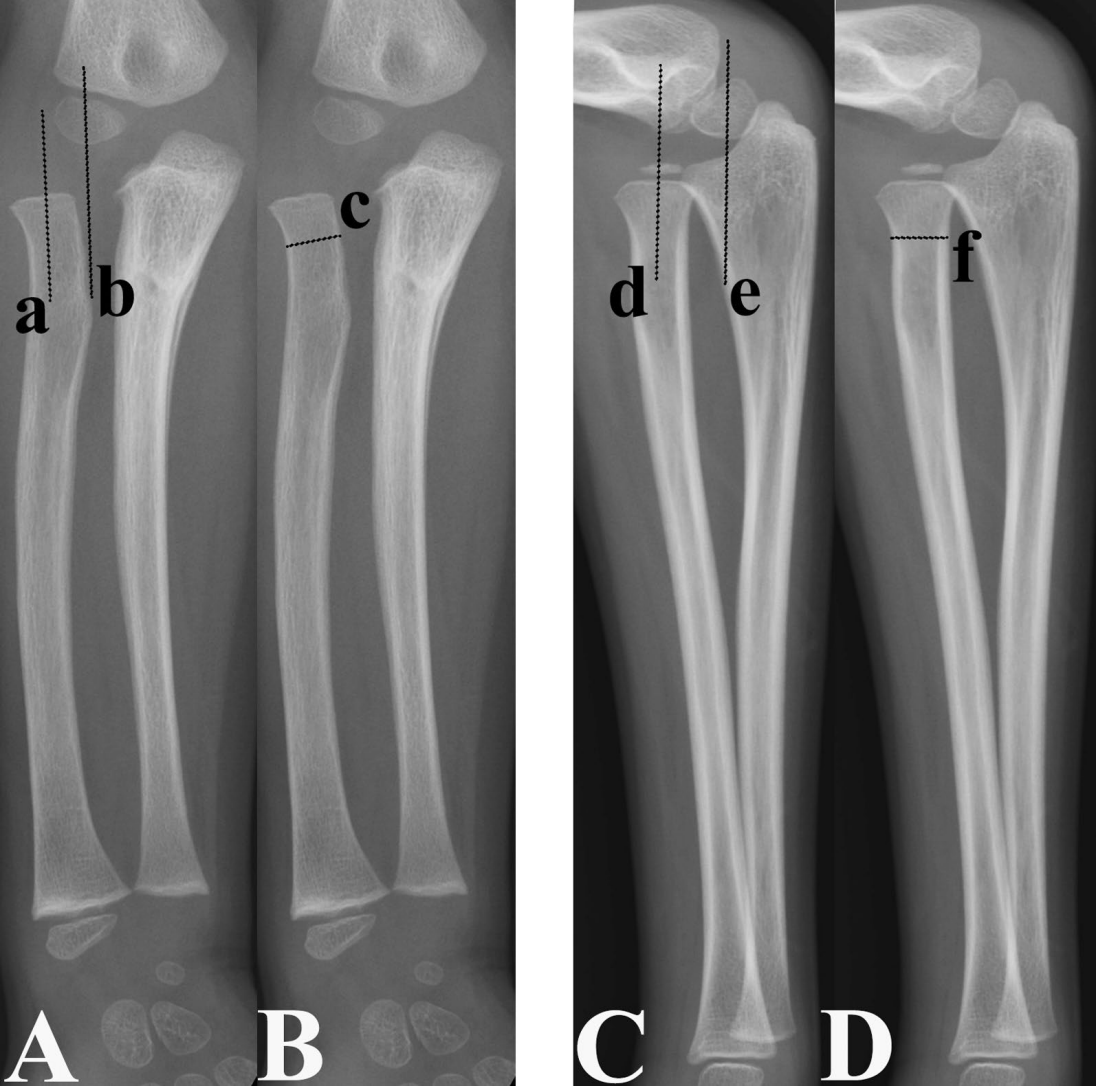
Measurement of the DD-RH on anteroposterior (**A** and **B**) and lateral (**C** and **D**) radiographs; DD-RH is expressed as the ratio of the distance between the forearm axes passing through the centre of the proximal radial metaphysis (a and d) and the centre of the humeral capitellum (b and e) to the narrowest radial neck width (lines c and f)

The distance of the dislocated RH from its anatomical position (DD-RH) was measured on preoperative AP and lateral radiographs (Fig. 2). Based on the measurement method reported by Wang et al. [14], the DD-RH is defined as the ratio of the distance between the forearm axes passing through the centre of the humeral capitellum and that of the proximal radial metaphysis related to the narrowest width of the radial neck. Higher DD-RH values on AP or lateral radiographs were used for further statistical analysis.

Two paediatric orthopaedists (W.W.T. and M.Q.Q.) independently measured the radiographic parameters twice at two-week intervals, and the mean values were recorded for statistical analysis. All the radiographic measurements were performed using the Picture Archiving and Communication Systems (PACS; GE, USA).

### Statistical analysis

Statistical analysis was performed with SPSS version 23.0 (SPSS Inc., Chicago, IL, USA). Data were expressed as numerical variables, frequencies and percentages with means and standard deviations. Paired-sample *t*-tests were performed to compare the differences in the RNS_AP_ angle and the RH_L_ size between the injured and uninjured elbow joints. According to the results of paired-sample *t* tests, single-factor analyses, including one-way analysis of variance (ANOVA), Student’s *t*-tests and Pearson correlation analyses were performed to evaluate the correlation between En-RSA_AP_ or En-RH_L_ and categorical variables (one-way ANOVA or Student’s *t*-tests), including institution, sex, laterality, direction of RH dislocation, presence of radial or median nerve injury, immobilization of the elbow joint and presence of heterotopic ossification, and continuous variables (Pearson correlation analysis), including age at the time of injury, time from injury to diagnosis and DD-RH. Then, according to the results of the single-factor analyses, factors with a *P* value < 0.1 were included in the multiple linear regression analysis. A *P* value < 0.05 indicated statistical significance.

## Results

Among the 142 patients with untreated CMFs, the mean ages at the time of injury and diagnosis and the time from injury to diagnosis were 5.73 ± 2.66 years (range, 1–14), 6.95 ± 2.83 years (range, 1–17) and 14.6 ± 21.55 months (range, 1–108), respectively.

### Comparison of the RNS_AP_ angle and RH_L_ size between the injured and uninjured sides

Paired-sample *t* tests revealed that the mean RH_L_ size of the injured elbows (1.43 ± 0.14, range: 1.18–1.94) was significantly greater than that (1.36 ± 0.1, range: 1.15–1.76) of the uninjured elbows (*t* = 6.49, *P* < 0.001); however, there were no significant differences in the RNS_AP_ angles between the injured elbows (13.22 ± 3.69°, range: 2.6–26°) and uninjured elbows (12.79 ± 3.29°, range: 2.7–27.2°) (*t* = 1.796, *P* = 0.075). The mean En-RNS_AP_, En-RH_L_ and DD-RH were 1.05 ± 0.24 (range, 0.44–1.9), 1.05 ± 0.09 (range, 0.89–1.3) and 1.34 ± 0.44 (range, 0.34–2.74), respectively.

### Risk factors for increased En-RH_L_ according to single-factor analysis

One-way ANOVA showed that En-RH_L_ was comparable among different institutions (*F* = 0.703, *P* = 0.842). The Pearson correlation analysis revealed that the En-RH_L_ and DD-RH increased significantly with the time from injury to diagnosis (*P* < 0.001), with correlation coefficients of 0.325 and 0.427, respectively. In addition, there was a slight correlation between En-RH_L_ and age at the time of injury according to the Pearson correlation analysis (correlation coefficient = 0.153, *P* = 0.069). Student’s *t*-test and one-way ANOVA did not reveal sex (*P* = 0.742), laterality (*P* = 0.113), direction of RH dislocation (*P* = 0.487), presence of radial or median nerve injury (*P* = 0.116), immobilization of the elbow joint after injury (*P* = 0.89) or presence of heterotopic ossification (*P* = 0.177) as risk factors for increased En-RH_L_ in patients with untreated CMFs (Table 1).

**Table 1:** Analysis of En-RH_L_ by patient demographics

		En-RH_L_ (%)	*t/F*	*P*
Sex	Female	1.04 ± 0.09	0.33	0.742
	Male	1.05 ± 0.08		
Laterality	Left	1.03 ± 0.08	1.593	0.113
	Right	1.06 ± 0.09		
Direction of RH dislocation	Anterior	1.06 ± 0.08	1.063	0.487
	Anterior-lateral	1.03 ± 0.09		
	Anterior-medial	1.08 ± 0.09		
	Lateral	1.01 ± 0.06		
	Posterior-lateral	1.02 ± 0.11		
Radial or median nerve injury	No	1.05 ± 0.09	1.581	0.116
	Yes	1.02 ± 0.07		
Immobilization of the elbow after injury	No	1.05 ± 0.1	0.138	0.89
	Yes	1.05 ± 0.07		
Heterotopic ossification	No	1.05 ± 0.09	1.399	0.177
	Yes	1.02 ± 0.07		

*RH* radial head, *En-RH*_*L*_ the ratio of the enlargement (En) of the RH_L_ size of the injured elbow to that of the uninjured elbow on lateral (L) radiographs

### Multiple linear regression analysis of the risk factors for increased En-RH_L_

**Fig. 3:**
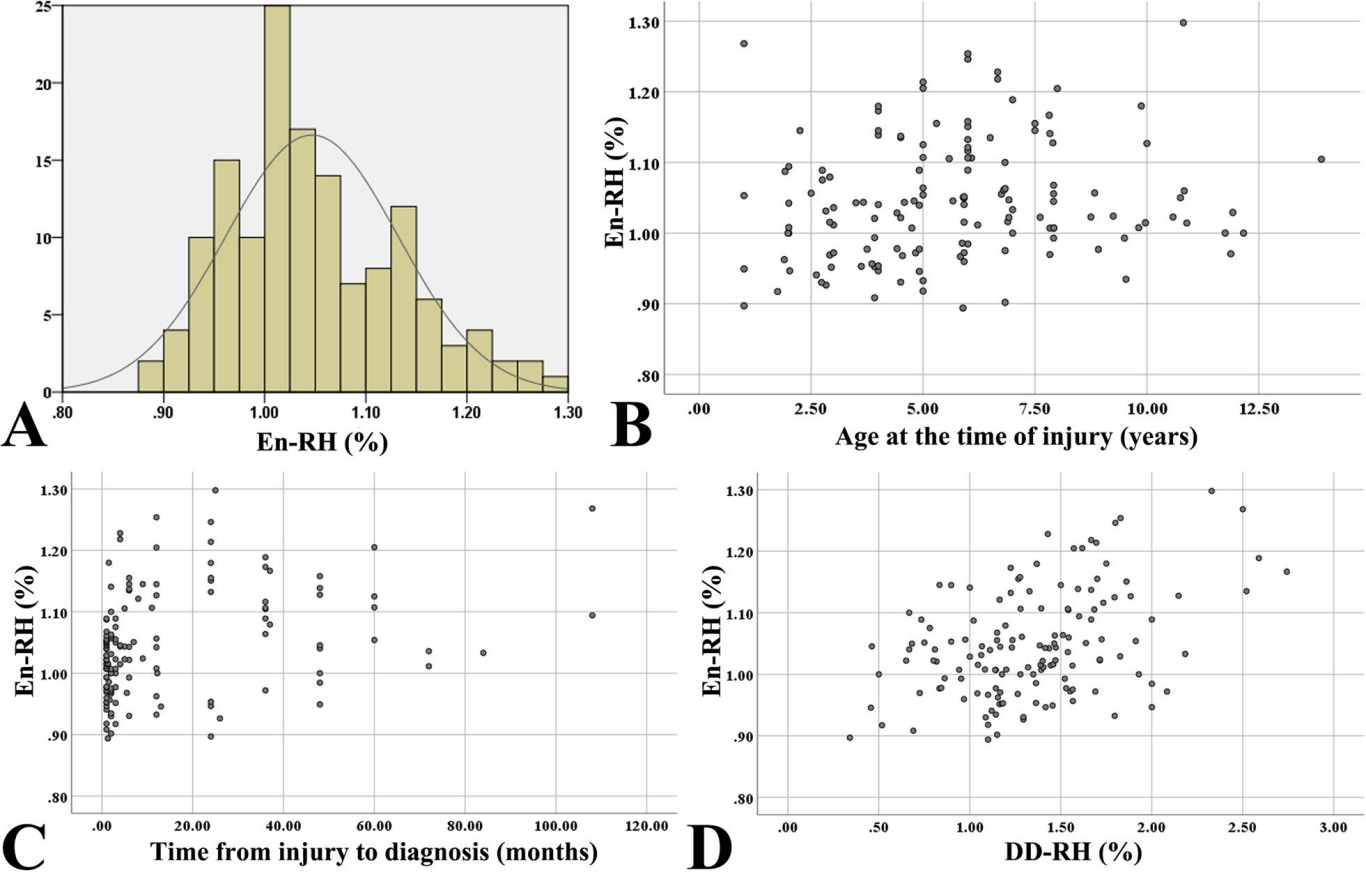
Correlation of normally distributed En-RH_L_ (**A**) with age at the time of injury (**B**), time from injury to diagnosis (**C**) and DD-RH (**D**)

According to the results described above, age at the time of injury, time from injury to diagnosis and DD-RH were included in the multiple linear regression analysis. Normality testing showed that En-RH_L_ was normally distributed (Fig. 3). ANOVA analysis revealed a successfully established regression model (*F* = 13.786, *P* < 0.001). Multiple linear regression analysis revealed that age at the time of injury (*P* = 0.047), time from injury to diagnosis (*P* = 0.007) and DD-RH (*P* = 0.001) were risk factors for increased En-RH_L_; the variability of En-RH_L_ among the three other risk factors was 21.4% (Table 2 and Fig. 3).

**Table 2:** Results of the multiple linear regression analysis

	*B*	*β*	*t*	*P*
Age at the time of injury	0.005	0.162	2.002	0.047*
Time from injury to diagnosis	0.001	0.238	2.715	0.007*
DD-RH	0.058	0.301	3.486	0.001*

*RH* radial head, *DD-RH* dislocation distance of radial head

^*^P < 0.05

## Discussion

The developmental pattern of PRA of the injured elbow in children with untreated CMFs has not been fully investigated in previous reports. In addition, the direct cause of anatomic reduction failure in paediatric patients with surgically treated CMFs is not known, although several risk factors for anatomic reduction failure have been reported [10–13]. In this study, we evaluated the PRA by comparing the differences in the RNS_AP_ angle and the RH_L_ size between injured and uninjured elbows in 142 paediatric patients with unilateral untreated CMFs. Then, single-factor analysis and multiple linear regression analysis were both used to investigate the risk factors for PRA of the injured elbows. Our results showed that the presence of PRA of the injured elbow in patients with untreated CMFs and several variables, including age at the time of injury, time from injury to diagnosis and DD-RH, were risk factors for this condition, which could also predict the failure of anatomical reduction in those with surgically treated CMFs according to previous studies [10–13]; consequently, PRA of the injured elbows could be considered as a direct cause of anatomical reduction failure in patients who underwent surgical treatment for CMFs. These findings confirmed our hypothesis.

**Fig. 4:**
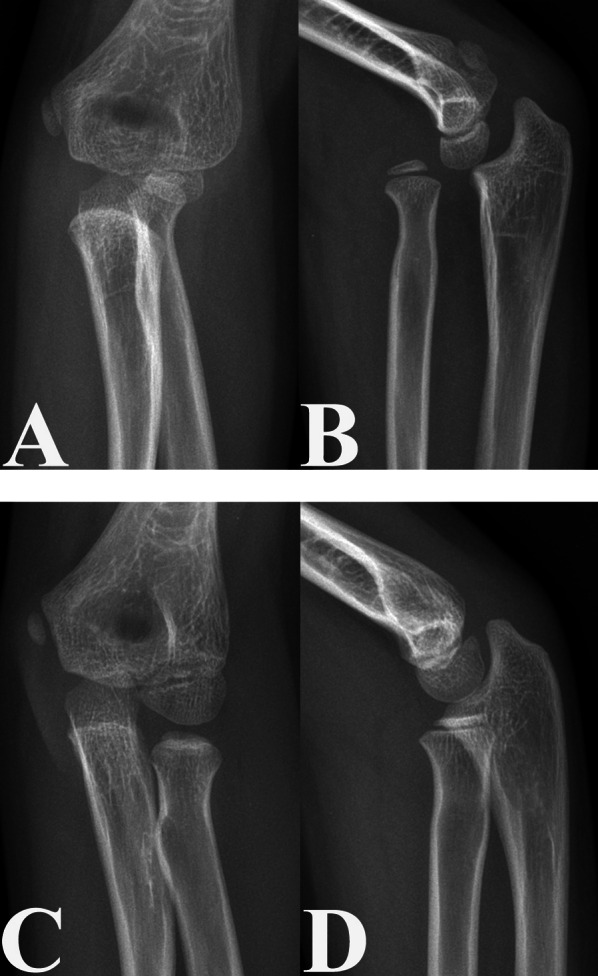
Anteroposterior (**A** and **C**) and lateral (**B** and **D**) radiographs of the injured (**A** and **B**) and uninjured (**C** and **D**) elbows in a 10-year-old boy with an untreated CMF, with a time from injury to diagnosis of 48 months and a DD-RH of 1.28%. The RNS_AP_ angles on anteroposterior radiographs of the injured (**A**) and uninjured (**C**) elbow joints were not significantly different

Interestingly, our study revealed that valgus deformities of the proximal radius were not present in the injured elbows of patients with untreated CMFs (Fig. 4). Our results differed from those reported by Kim et al. [9] and Baydar et al. [7]. Kim et al. reviewed 12 unilateral paediatric CMFs and reported that the RNS_AP_ angle of the injured elbow was greater than that of the uninjured elbow [9]. Baydar et al. also reported similar findings [7]. This discrepancy may be due to the lack of statistical analysis in their studies [7, 9]. In this study, the mean RNS_AP_ angle of the injured elbows was greater than that of the uninjured elbows, but Student’s *t*-test revealed no significant differences in the RNS_AP_ angles between the injured and uninjured elbows (*P* = 0.075). In addition, the sample size in our study (*n* = 142) was larger than that in the studies reported by Kim et al. (*n* = 12) and Baydar et al. (*n* = 14), thereby strengthening the statistical power of our results [7, 9].

The valgus deformities of the proximal radius are generally caused by arrested growth of the lateral portion of the proximal radial physis [18]. However, Monteggia fractures in paediatric patients are not associated with injury to the proximal radial growth plate according to Bado’s classification system [1]. Furthermore, patients with concomitant fracture of the proximal radial growth plate were excluded in this study. In addition, disruption of the vessels supplying the proximal radius after trauma affects the growth of the entire proximal radial physis, not just the lateral portion of the growth plate [19]. Therefore, the patients with untreated CMFs in this study did not develop valgus deformities of the proximal radius after injury.

In this study, we found that the En-RH_L_ was increased in the injured elbows of patients with untreated CMFs. Our results confirmed the intraoperative observations reported in previous studies that revealed an enlarged RH in paediatric patients with CMFs, although those studies lacked radiographic measurements and statistical analyses [7, 8]. In addition, Kim et al. reported similar results using the same method of radiographic measurement to evaluate RH_L_ size in 12 children with CMFs [9]. They reported that the RH_L_ size of the injured elbow was greater than that of the uninjured elbow, although no statistical analysis was performed [9]. Due to accurate radiographic measurements and sufficient statistical analysis of an adequate sample size (*n* = 142), our results had sufficient statistical power.

**Fig. 5:**
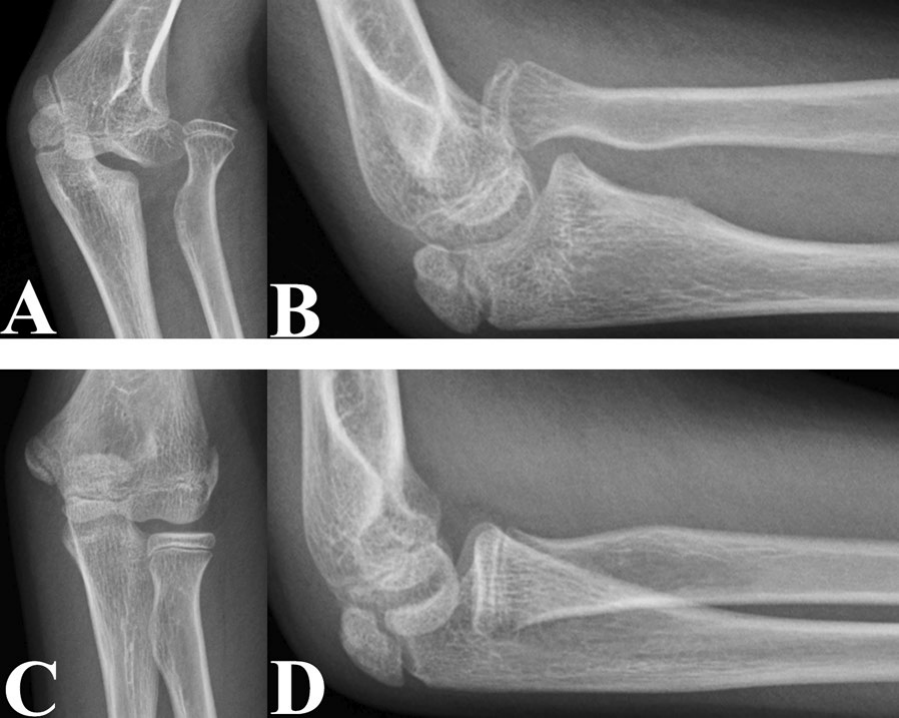
Anteroposterior (**A** and **C**) and lateral (**B** and **D**) radiographs of the injured (**A** and **B**) and uninjured (**C** and **D**) elbows in a 12-year-old boy with an untreated CMF, a time from injury to diagnosis of 25 months, and a DD-RH of 2.33%. Lateral radiographs showed a greater En-RH_L_ in the injured elbow (**B**) than in the uninjured elbow (**D**)

**Fig. 6:**
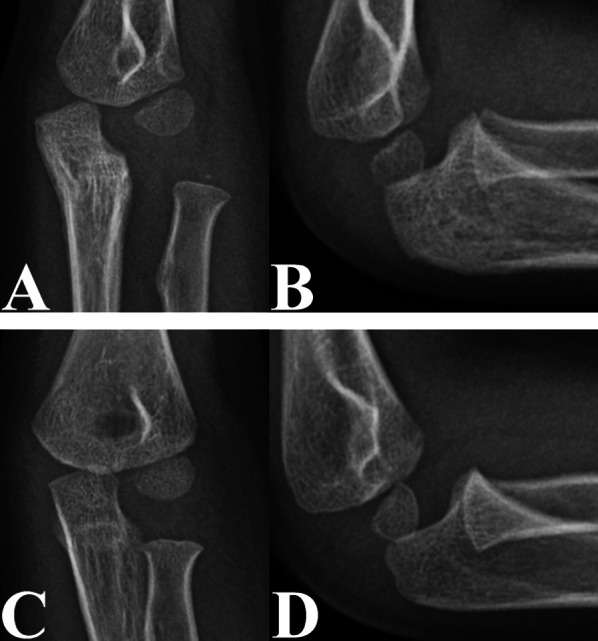
Anteroposterior (**A** and **C**) and lateral (**B** and **D**) radiographs of the injured (**A** and **B**) and uninjured (**C** and **D**) elbows in a 5-year-old boy with an untreated CMF, a time from injury to diagnosis of 1 month, and a DD-RH of 0.73%. Lateral radiographs showed no significant difference in the En-RH_L_ between the injured (**B**) and uninjured (**D**) elbow joints

Our study also revealed that older age at the time of injury was associated with a greater En-RH_L_ in patients with untreated CMFs (Figs. 5 and 6). This finding may be explained by the increased growth capacity of older children. Several reports have shown that the growth capacity of the physis increases with age prior to closure of the Y-cartilage, which occurs between 12 and 14 years of age [20, 21]. Furthermore, the mean age at the time of diagnosis of CMFs in our study was 6.95 years, indicating that the growth plates were still open in these patients. Consequently, in the microenvironment that induces RH enlargement, older paediatric patients with CMFs exhibit a greater En-RH_L_ due to increased growth potential.

In addition, our data identified an increased DD-RH as a risk factor for a greater En-RH_L_ caused by RH enlargement or radial neck narrowing (Figs. 5 and 6). These results could be explained by the disruption of the vessels supplying the proximal radius after trauma, especially in patients with a larger DD-RH. Several studies have shown that PRA after injury is mainly caused by disruption of the vascular supply to the proximal radius [22–24]. In addition, these results might be due to hypoxia and ischaemia being better tolerated by the growth plate than bone. Previous reports have shown that hypoxia and ischaemia suppress osteogenesis [25–28]. Therefore, it is likely that abnormal growth is more likely to occur in the radial neck after the disruption of nutrient vessels, resulting in an abnormal radial neck shape and size [25–28]. In addition, patients with a larger DD-RH are likely to have significant damage to the capsule and annular ligaments, which are the main stabilizers of the RH [29, 30]; consequently, the dislocated RH was more unstable in CMFs with a larger DD-RH [29, 30]. Due to the instability of the dislocated RH, the proximal radial growth plate can be stimulated by more abnormal mechanical forces, which can stimulate bone formation [31, 32]. Therefore, an increased DD-RH was considered a risk factor for a greater En-RH_L_ in patients with untreated CMFs.

Our study also showed that the time from injury to diagnosis was a risk factor for an increased En-RH_L_ in patients with untreated CMFs and that the severity of PRA of the injured elbow increased with the time from injury to diagnosis (Figs. 5 and 6). Our results were consistent with those reported by Kim et al. [9]. These results further supported our hypothesis that PRA in patients with untreated CMFs was mainly caused by the disruption of the vessels supplying the proximal radius and the mechanical stimulation on the proximal radial physis. According to several previous reports, pathological changes in bone tissues after disruption of nutrient vessels are gradually apparent on radiographs [33, 34]. In addition, other studies revealed that the osteogenic capacity increased with the duration of mechanical stimulation [35, 36]. Therefore, PRA tends to be more severe in patients with untreated paediatric CMFs, especially those with longer times to diagnosis and subsequent treatment.

Notably, our study has several limitations. First, this was a retrospective study. Second, only two radiographic parameters (RNS_AP_ angle and RH_L_ size), which have been widely used in previous studies, were measured to reflect the severity of PRA [9, 17, 37]. Third, the detailed molecular mechanism underlying the increase in En-RH_L_ was not elucidated. Fourth, the surgical management of PRA was not reported and the potential correlation between surgical procedures and PRA was not evaluated. Despite these limitations, to our knowledge, this was the first study in which the radiographic changes associated with PRA were evaluated in an adequately sized sample of children with untreated CMFs and a systematic analysis was performed to investigate the risk factors for PRA. Most importantly, our study revealed a significant correlation between PRA of the injured elbow and several risk factors, including age at the time of injury, time from injury to diagnosis, and DD-RH.

In conclusion, in paediatric patients with untreated CMFs, PRA of the injured elbow mainly consisted of enlargement of the RH or narrowing of the radial neck rather than valgus deformities of the proximal radius. Older age at the time of injury, increased time from injury to diagnosis, and DD-RH were identified as risk factors for a greater En-RH_L_ of the injured elbows in patients with untreated CMFs. Therefore, more severe PRA could be considered a direct cause of surgical reduction failure in paediatric patients with CMFs. Additional prospective, randomized clinical trials and molecular and biological experiments are needed to confirm our findings.

## Data Availability

The datasets generated and analysed in the current study are not publicly available due to data protection regulations. Access to data is limited to the researchers who have obtained permission for data processing. Further inquiries can be made to the corresponding author.

## Abbreviations

PRA: Proximal radial abnormalities
CMFs: Chronic monteggia fractures
RNS: Radial neck-shaft
RH: Radial head
DD-RH: Dislocation distance of radial head
AP: Anteroposterior
L: Lateral
En: Enlargement

## References

[1] Bado JL (1967) The Monteggia lesion. Clin Orthop Relat Res 50:71–86 6029027

[2] Kay RM, Skaggs DL (1998) The pediatric Monteggia fracture. Am J Orthop 27:606–609 9758451

[3] Bae DS (2016) Successful strategies for managing Monteggia injuries. J Pediatr Orthop 36(Suppl 1):S67–S70. 10.1097/BPO.0000000000000765 27100040 10.1097/BPO.0000000000000765

[4] Goyal T, Arora SS, Banerjee S, Kandwal P (2015) Neglected Monteggia fracture dislocations in children: a systematic review. J Pediatr Orthop B 24:191–199. 10.1097/BPB.0000000000000147 25714935 10.1097/BPB.0000000000000147

[5] Liao S, Pan J, Lin H, Xu Y, Lu R, Wu J et al (2019) A new approach for surgical treatment of chronic Monteggia fracture in children. Injury 50:1237–1241. 10.1016/j.injury.2019.04.017 31056214 10.1016/j.injury.2019.04.017

[6] Mohan Kumar EG, Yathisha Kumar GM, Noorudheen M (2019) Functional outcome of bell tawse procedure for the management of chronic unreduced Monteggia fracture-dislocation in children. Indian J Orthop 53:745–750. 10.4103/ortho.IJOrtho_47_19 31673176 10.4103/ortho.IJOrtho_47_19 PMC6804388

[7] Baydar M, Öztürk K, Orman O, Akbulut D, Keskinbıçkı MV, Şencan A (2022) Use of corrective ulnar osteotomy and radial head relocation into preserved annular ligament in the treatment of radiocapitellar instability secondary to pediatric chronic Monteggia fracture-dislocation. J Hand Surg Am 47:481.e1-481.e9. 10.1016/j.jhsa.2021.05.025 34253391 10.1016/j.jhsa.2021.05.025

[8] Stragier B, De Smet L, Degreef I (2018) Long-term follow-up of corrective ulnar osteotomy for missed Monteggia fractures in children. J Shoulder Elbow Surg 27:e337–e343. 10.1016/j.jse.2018.06.029 30224208 10.1016/j.jse.2018.06.029

[9] Kim HT, Conjares JN, Suh JT, Yoo CI (2002) Chronic radial head dislocation in children, part 1: pathologic changes preventing stable reduction and surgical correction. J Pediatr Orthop 22:583–590 12198458

[10] Nakamura K, Hirachi K, Uchiyama S, Takahara M, Minami A, Imaeda T et al (2009) Long-term clinical and radiographic outcomes after open reduction for missed Monteggia fracture-dislocations in children. J Bone Joint Surg Am 91:1394–1404. 10.2106/JBJS.H.00644 19487517 10.2106/JBJS.H.00644

[11] Rahbek O, Deutch SR, Kold S, Søjbjerg JO, Møller-Madsen B (2011) Long-term outcome after ulnar osteotomy for missed Monteggia fracture dislocation in children. J Child Orthop 5:449–457. 10.1007/s11832-011-0372-0 23205146 10.1007/s11832-011-0372-0 PMC3221759

[12] Ko KR, Shim JS, Park J, Won J (2022) Predictors of ideal outcomes after reconstructive surgery for chronic Monteggia fracture in children. J Orthop Sci 27:1025–1031. 10.1016/j.jos.2021.06.009 34452791 10.1016/j.jos.2021.06.009

[13] Cao S, Dong ZG, Liu LH, Wei JW, Luo ZB, Peng P (2022) Missed Monteggia fractures in children treated by open reduction of the radial head and corrective osteotomy of the ulna. Sci Rep 12:16819. 10.1038/s41598-022-21019-4 36207388 10.1038/s41598-022-21019-4 PMC9546922

[14] Wang W, Xiong Z, Huang D, Li Y, Huang Y, Guo Y et al (2024) Risk factors for unsuccessful reduction of chronic Monteggia fractures in children treated surgically: a review of 209 cases. Bone Jt Open 5:581–591. 10.1302/2633-1462.56.BJO-2024-0004.R2 38991554 10.1302/2633-1462.57.BJO-2024-0004.R2 PMC11247538

[15] Meyers C, Lisiecki J, Miller S, Levin A, Fayad L, Ding C et al (2019) Heterotopic ossification: a comprehensive review. JBMR Plus 3:e10172. 10.1002/jbm4.10172 31044187 10.1002/jbm4.10172 PMC6478587

[16] Foruria AM, Augustin S, Morrey BF, Sánchez-Sotelo J (2013) Heterotopic ossification after surgery for fractures and fracture-dislocations involving the proximal aspect of the radius or ulna. J Bone Joint Surg Am 95:e66. 10.2106/JBJS.K.01533 23677367 10.2106/JBJS.K.01533

[17] Kim HT, Can LV, Ahn TY, Kim IH (2017) Analysis of radiographic parameters of the forearm in traumatic radial head dislocation. Clin Orthop Surg 9:521–528. 10.4055/cios.2017.9.4.521 29201306 10.4055/cios.2017.9.4.521 PMC5705312

[18] Arrigoni C, Catena N (2022) Chronic Monteggia in pediatric population: a narrative literature review. Pediatr Med Chir. 10.4081/pmc.2022.289.10.4081/pmc.2022.289 37184321 10.4081/pmc.2022.289

[19] Young S, Letts M, Jarvis J (2000) Avascular necrosis of the radial head in children. J Pediatr Orthop 20:15–18 10641681

[20] Dimeglio A (2001) Growth in pediatric orthopaedics. J Pediatr Orthop 21:549–555 11433174

[21] Ducan B, Lubchenco LO, Hansman C (1974) Growth charts for children 0 to 18 years of age. Pediatrics 54:497–502 4413606

[22] Kalbitz M, Lackner I, Perl M, Pressmar J (2023) Radial head and neck fractures in children and adolescents. Front Pediatr 10:988372. 10.3389/fped.2022.988372 36741096 10.3389/fped.2022.988372 PMC9897312

[23] Hell AK, von Laer L (2014) Wachstumsverhalten nach frakturen des proximalen radiusendes: unterschiede zum übrigen skelett [growth behaviour after fractures of the proximal radius: differences to the rest of the skeleton]. Unfallchirurg 117:1085–1091. 10.1007/s00113-014-2632-1 25427529 10.1007/s00113-014-2632-1

[24] Vocke AK, von Laer LR (1998) Die prognose proximaler radiusfrakturen im wachstumsalter [prognosis of proximal radius fractures in the growth period]. Unfallchirurg 101:287–295. 10.1007/s001130050270 9613214 10.1007/s001130050270

[25] Schipani E, Ryan HE, Didrickson S, Kobayashi T, Knight M, Johnson RS (2001) Hypoxia in cartilage: HIF-1alpha is essential for chondrocyte growth arrest and survival. Genes Dev 15:2865–2876. 10.1101/gad.934301 11691837 10.1101/gad.934301 PMC312800

[26] Brucker PU, Izzo NJ, Chu CR (2005) Tonic activation of hypoxia-inducible factor 1alpha in avascular articular cartilage and implications for metabolic homeostasis. Arthritis Rheum 52:3181–3191. 10.1002/art.21346 16200622 10.1002/art.21346

[27] Arnett TR (2010) Acidosis, hypoxia and bone. Arch Biochem Biophys 503:103–109. 10.1016/j.abb.2010.07.021 20655868 10.1016/j.abb.2010.07.021

[28] Xu D, Gan K, Wang Y, Wu Z, Wang Y, Zhang S et al (2022) A composite deferoxamine/black phosphorus nanosheet/gelatin hydrogel scaffold for ischemic tibial bone repair. Int J Nanomedicine 17:1015–1030. 10.2147/IJN.S351814 35299865 10.2147/IJN.S351814 PMC8923703

[29] Galik K, Baratz ME, Butler AL, Dougherty J, Cohen MS, Miller MC (2007) The effect of the annular ligament on kinematics of the radial head. J Hand Surg Am 32:1218–1224. 10.1016/j.jhsa.2007.06.008 17923306 10.1016/j.jhsa.2007.06.008

[30] Xu G, Chen W, Yang Z, Yang J, Liang Z, Li W (2022) Finite element analysis of elbow joint stability by different flexion angles of the annular ligament. Orthop Surg 14:2837–2844. 10.1111/os.13452 36106628 10.1111/os.13452 PMC9627061

[31] Li X, Han L, Nookaew I, Mannen E, Silva MJ, Almeida M et al (2019) Stimulation of piezo1 by mechanical signals promotes bone anabolism. Elife 8:e49631. 10.7554/eLife.49631 31588901 10.7554/eLife.49631 PMC6779475

[32] Rosa N, Simoes R, Magalhães FD, Marques AT (2015) From mechanical stimulus to bone formation: a review. Med Eng Phys 37:719–728. 10.1016/j.medengphy.2015.05.015 26117332 10.1016/j.medengphy.2015.05.015

[33] Wang WT, Li YQ, Guo YM, Li M, Mei HB, Shao JF et al (2019) Risk factors for the development of avascular necrosis after femoral neck fractures in children: a review of 239 cases. Bone Joint J 101-B:1160–1167. 10.1302/0301-620X.101B9.BJJ-2019-0275.R1 31474136 10.1302/0301-620X.101B9.BJJ-2019-0275.R1

[34] Wang W, Li Y, Guo Y, Li M, Mei H, Shao J et al (2020) Initial displacement as a risk factor for avascular necrosis of the femoral head in pediatric femoral neck fractures: a review of one hundred eight cases. Int Orthop 44:129–139. 10.1007/s00264-019-04429-4 31655884 10.1007/s00264-019-04429-4

[35] Zhang P, Liu X, Guo P, Li X, He Z, Li Z et al (2021) Effect of cyclic mechanical loading on immunoinflammatory microenvironment in biofabricating hydroxyapatite scaffold for bone regeneration. Bioact Mater 6:3097–3108. 10.1016/j.bioactmat.2021.02.024 33778191 10.1016/j.bioactmat.2021.02.024 PMC7960680

[36] Wu J, Tang Y, Pu X, Wang M, Chen F, Chen X et al (2021) The role of micro-vibration parameters in inflammatory responses of macrophages cultured on biphasic calcium phosphate ceramics and the resultant influence on osteogenic differentiation of mesenchymal stem cells. J Mater Chem B 9:8003–8013. 10.1039/d1tb00898f 34476430 10.1039/d1tb00898f

[37] Delpont M, Jouve JL, de Sales Gauzy J, Louahem D, Vialle R, Bollini G et al (2014) Proximal ulnar osteotomy in the treatment of neglected childhood Monteggia lesion. Orthop Traumatol Surg Res 100:803–807. 10.1016/j.otsr.2014.06.022 25304829 10.1016/j.otsr.2014.06.022

